# Immune-mediated resolution of a herniated lumbar disc prolapse aided by motor control exercises—a case report

**DOI:** 10.1093/jscr/rjad644

**Published:** 2023-12-05

**Authors:** Abigail J Garcia, George Ampat

**Affiliations:** University of the Incarnate Word School of Osteopathic Medicine, San Antonio, TX 78235, United States; School of Medicine, University of Liverpool, Cedar House, Ashton St, Liverpool L69 3GE, United Kingdom

**Keywords:** low back pain, intervertebral disc displacement, exercise therapy, immune privilege

## Abstract

Low back pain is the global leading cause of disability. Herniated intervertebral discs are a common cause of lower back pain. The natural history of the herniated intervertebral disc is that it can resorb spontaneously through an immune-mediated mechanism. Despite this favourable natural history, there is an increasing reliance on surgical intervention. A 64-year-old presented with a left L3/4 disc prolapse. With reassurance, simple analgesics, and motor control exercises, the MRI scan confirmed the complete resolution of the disc prolapse within 3 months. Patients with disc prolapses should be reassured that disc prolapses will naturally resolve and advised to remain active. Surgical intervention should only be considered with the presence of red flags, progressive neurology, or when clear evidence exists that all non-interventional techniques have been exhausted. With such a favourable natural history, caution should be exercised before surgical intervention is recommended.

## Introduction

Low back pain (LBP) is the global leading cause of years lived with disability [1]. Whilst the age-standardized point prevalence of LBP decreased from 1990 to 2017, the years lived with disability increased significantly [1]. LBP accompanied by neurological deficits and radiculopathy symptoms suggests possible damage to neural tissues by a herniated intervertebral disc. Lumbar discectomy has been shown to be superior to non-operative management in the Spine Patient Outcomes Research Trial in carefully selected patients with disc herniation [2]. However, the same trial reported that non-operatively treated patients also achieved substantial improvements over time [3]. Even though most disc prolapses resolve naturally the rates of spine surgeries being performed are increasing [4]. Unfortunately, spinal surgery is associated with an increased risk of recurrent LBP [5], recurrent herniations, postoperative degeneration, and failed back surgery syndrome [6]. The increased level of spinal surgeries may be because of the paucity of information in the public domain about the immune-mediated resorption of the prolapsed disc [7] and the lack of knowledge regarding the appropriate exercise required to deal with disc herniations [8]. Here, we present a case of a patient with radicular LBP and L3/L4 disc herniation that resolved non-operatively with motor control exercises.

## Case report

**Figure 1:**
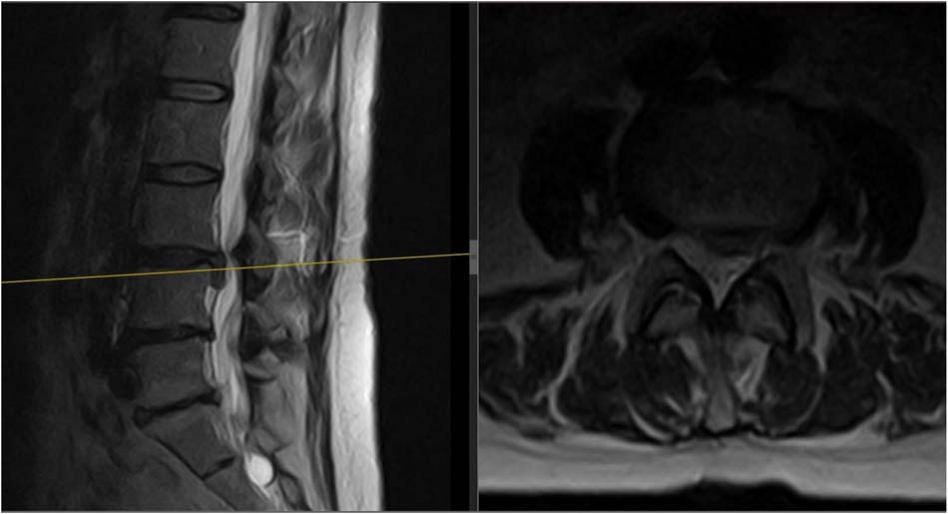
Initial MRI scan of the lumbar spine: initial MRI scan of the lumbar spine performed in July 2022 showing the left-sided L3/4 disc prolapse.

**Figure 2:**
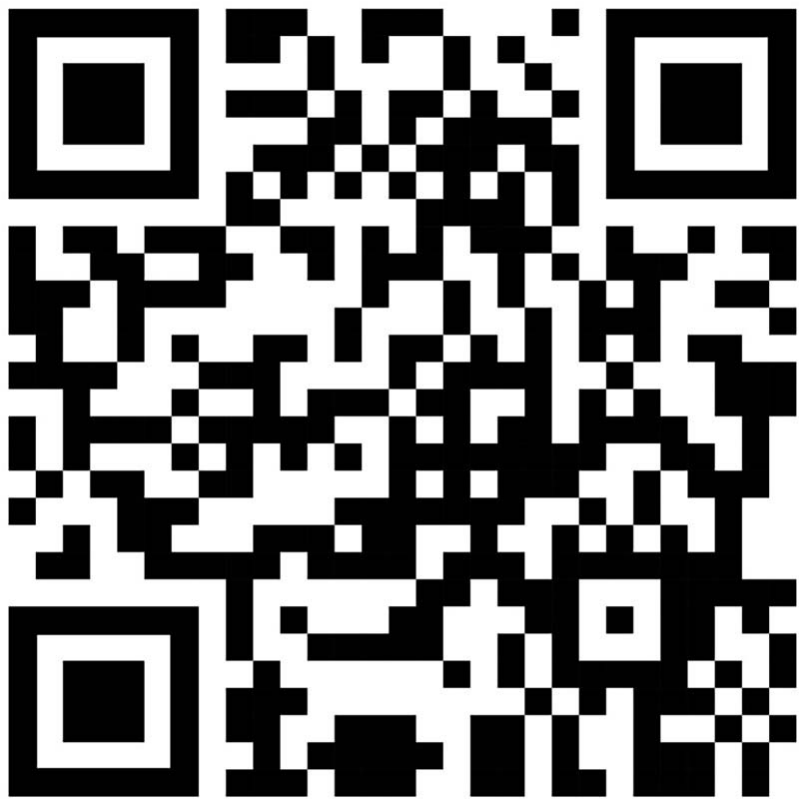
QR code providing the YouTube video by the senior author detailing the motor control exercises for lower back pain.

A 64-year-old female secretary presented with severe LBP and left anterior thigh pain that began 6 weeks prior to presentation whilst cleaning her house. She had no loss of bowel or bladder control, saddle anaesthesia, unintentional weight loss, or loss of appetite. A physical exam revealed decreased forward flexion, intact sensation, and intact motor function of the lower extremities. There was some tenderness in the left hip and gluteal regions. The straight leg raise was negative on the left but the femoral stretch test was mildly positive. Initial imaging showed an L3/L4 central and left paracentral disc extrusion with compression of the left L4 transiting nerve roots (Fig. 1). Benign haemangiomas were present at L2 and L4, along with a benign perineural cyst at S2. The patient was reassured and shown how to perform the motor control exercise programme as modified by the senior author (https://youtu.be/xJiAqVsfpRc) (Fig. 2).

The goal of this exercise programme is to train the multifidus muscle to activate synchronously with the other muscles and thereby increase the stability of the spine. For pain relief, the patient was advised to take paracetamol and codeine.

**Figure 3:**
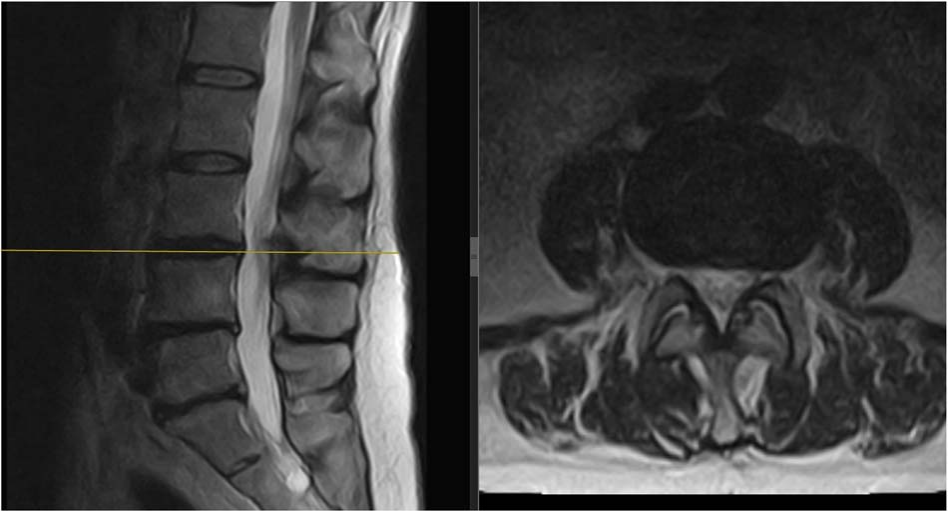
Follow-up MRI scan of the lumbar spine: follow-up MRI scan of the lumbar spine performed in March 2023 showing complete resolution of the disc prolapse.

Three months later, the patient noted significant pain reduction. A physical exam revealed an increased range of motion with the patient able to reach her toes whilst flexing forward. No neurological deficits were present. Repeat MRIs showed improvement and resorption of the disc material at L3/L4, relieving the compression on the L4 nerve root (Fig. 3).

## Discussion

Spontaneous regression of a herniated intervertebral disc occurs through an immune-mediated mechanism [7]. The intervertebral disc derives embryologically from the notochord and sclerotome. The notochord develops into the nucleus pulposus (NP) and the sclerotome-derived connective tissue envelops it and forms the annulus fibrosus and vertebrae. Hence, during development, the annulus fibrosus that surrounds and the two cartilaginous endplates on either end enclose the NP, and that forms the blood–NP barrier [9]. The NP remains avascular. The avascularity of the NP and the blood–NP barrier shields the NP from the host immune system. Because of the presence of the blood–NP barrier, the immune system does not develop self-tolerance to the NP [9]. Therefore, when the NP herniates into the epidural space, it triggers an immune reaction characterized by infiltration of macrophages, lymphocytes, natural killer cells, and mast cells [8]. In addition, these cells trigger the initiation of the inflammatory cascade and the release of inflammatory factors such as phospholipase A2, leukotrienes, and fibroblast growth factor. Matrix remodelling and neovascularisation are also determinants of spontaneous regression of the disc [9]. These processes collectively contribute to the degradation and resorption of the disc.

However, despite the natural resolution of a prolapsed disc, the rate of spinal surgeries performed in the USA is increasing [4]. This increased rate could possibly be because of the paucity of publicly available information about the immune-mediated resorption of prolapsed discs and the lack of common knowledge of the appropriate exercise to perform in acute disc prolapses.

Motor control training is a recognized method of exercise therapy for herniated lumbar intervertebral discs [8]. The motor control training programme retrains the core musculature to fire synchronously and improve segmental support [10].

**Figure 4:**
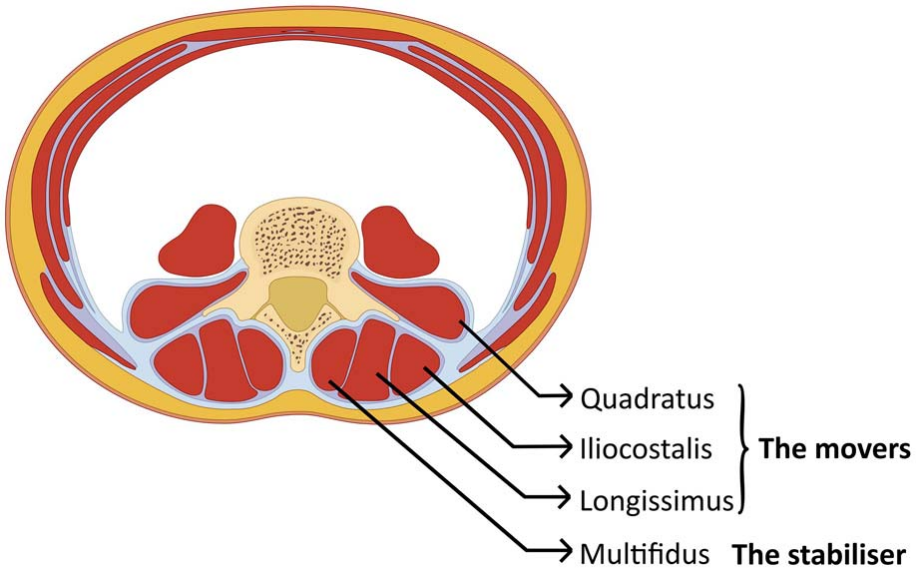
Schematic cross-section of the abdomen: showing the vertebra in the back with the muscles that surround the spine. The deepest layer is the uni-segmental multifidus (the stabilizers), which needs to activate before the multisegmental muscles in the outer layer (the movers).

Spinal muscles can be categorized into two groups: the deep, uni-segmental multifidus muscles that provide stability, and the larger multisegmented erector spinae muscles responsible for movement (Fig. 4). During normal spinal motion, the stabilizers should activate before the movers. However, in low back disorders, there is a loss of synchrony and reduced multifidus muscle activity, leading to compensatory actions by the erector spinae muscles, which can increase pressure on the intervertebral discs and cause pain during exercise [10].

To address this issue, a motor control training programme is used, consisting of three phases: synchrony, stability, and strengthening. In the synchrony phase, the abdominal drawing-in manoeuvre is performed in different positions to retrain the deep multifidus to activate before the larger erector spinae. The stability phase involves performing the abdominal drawing-in manoeuvre to train the multifidus during limb movement. Finally, during the strengthening phase, the abdominal drawing-in manoeuvre is performed whilst gradually increasing the endurance strength of the core muscles. This staged approach can improve compliance and support natural resolution of the prolapsed disc. These exercises may enhance immune-mediated disc resorption by increasing blood flow to the area. Yet, the precise connection between motor control exercises and this process is unclear, requiring further research to understand how exercise influences immune-mediated disc resorption.
